# The Vital Role of Central Executive Network in Brain Age: Evidence From Machine Learning and Transcriptional Signatures

**DOI:** 10.3389/fnins.2021.733316

**Published:** 2021-09-07

**Authors:** Keke Fang, Shaoqiang Han, Yuming Li, Jing Ding, Jilian Wu, Wenzhou Zhang

**Affiliations:** ^1^Department of Pharmacy, Affiliated Cancer Hospital of Zhengzhou University, Henan Cancer Hospital, Zhengzhou, China; ^2^Department of Magnetic Resonance Imaging, The First Affiliated Hospital of Zhengzhou University, Zhengzhou, China; ^3^Department of Radiotherapy, Affiliated Cancer Hospital of Zhengzhou University, Henan Cancer Hospital, Zhengzhou, China

**Keywords:** brain age, Allen Human Brain Atlas, structural brain imaging, machine learning, gene

## Abstract

Recent studies combining neuroimaging with machine learning methods successfully infer an individual’s brain age, and its discrepancy with the chronological age is used to identify age-related diseases. However, which brain networks play decisive roles in brain age prediction and the underlying biological basis of brain age remain unknown. To answer these questions, we estimated an individual’s brain age in the Southwest University Adult Lifespan Dataset (*N* = 492) from the gray matter volumes (GMV) derived from T1-weighted MRI scans by means of Gaussian process regression. Computational lesion analysis was performed to determine the importance of each brain network in brain age prediction. Then, we identified brain age-related genes by using prior brain-wide gene expression data, followed by gene enrichment analysis using Metascape. As a result, the prediction model successfully inferred an individual’s brain age and the computational lesion prediction results identified the central executive network as a vital network in brain age prediction (Steiger’s *Z* = 2.114, *p* = 0.035). In addition, the brain age-related genes were enriched in Gene Ontology (GO) processes/Kyoto Encyclopedia of Genes and Genomes (KEGG) pathways grouped into numbers of clusters, such as regulation of iron transmembrane transport, synaptic signaling, synapse organization, retrograde endocannabinoid signaling (e.g., dopaminergic synapse), behavior (e.g., memory and associative learning), neurotransmitter secretion, and dendrite development. In all, these results reveal that the GMV of the central executive network played a vital role in predicting brain age and bridged the gap between transcriptome and neuroimaging promoting an integrative understanding of the pathophysiology of brain age.

## Introduction

Normal brain aging is accompanied by a decline of brain region volumes (Anderton, 2002) and cognition such as conceptual reasoning, executive function, and memory (Harada et al., 2013; Kirova et al., 2015). As the brain ages, many age-related diseases emerge, such as Alzheimer’s disease (AD) (Amaducci and Tesco, 1994; Ferri et al., 2005). As the fifth leading cause of death in people over the age of 65 years (Kirova et al., 2015), AD burdens the society heavily. The risk of developing AD increases exponentially with age (Plassman et al., 2007). Thus, revealing the mechanism of the normal brain age is the key to understanding age-related diseases (Raji et al., 2009). Recent studies combining neuroimaging and machine learning methods predict brain age successfully and found that the chronological age is not exactly equal to brain age in both normal and pathological subjects such as patients with schizophrenia, mild cognitive impairments, and depression (Gaser et al., 2013; Habes and Janowitz, 2016; Hajek et al., 2019; Han et al., 2021; He et al., 2020). This discordance between brain age and chronological age helps explain individual differences in brain aging (Jylhävä et al., 2017). However, the underlying biological basis of brain age is not well elaborated.

Extensive efforts have been made to identify reliable indictors of biological age (Wagner et al., 2016). In recent years, the brain age method identifying normal aging pattern has turned out to be an informative biomarker of healthy brain aging at the individual level (Cole and Franke, 2017; Franke et al., 2010). For example, Vishnu et al. accurately predicted MRI-derived brain age, helping to identify various brain diseases (Bashyam et al., 2020). Using this framework, studies have uncovered accelerated brain aging in several neurological diseases using the brain-predicted age difference (brain-PAD) scores, defined as the discordance between the predicted brain age and the chronological age (Gaser et al., 2013; Habes and Janowitz, 2016; Hajek et al., 2019; Han et al., 2021; He et al., 2020). The brain age method outperforms other state-of-the-art biomarkers, with accuracy rates reaching 81% in identifying mild cognitive impairments (Gaser et al., 2013). Despite these remarkable findings, these studies have failed to elucidate the underlying biological basis of brain age, limiting our understanding of the biological mechanism of brain age and its application.

It is widely accepted that genetic factors play important roles in normal brain aging (Lin et al., 2020). For example, the expressions of genes playing roles in synaptic functional and neuronal plasticity in the frontal cortex are reduced with aging (Sikora et al., 2021). However, the relation between genetic factors and brain age derived from neuroimaging remains unknown. Advances in comprehensive brain-wide gene expression atlases make possible linking the spatial variations in gene expressions to macroscopic neuroimaging phenotypes (Fornito et al., 2019; Zhu et al., 2021). For example, Reardon et al. found that the genetic spatial expression is tied with cortical scaling gradients (Reardon and Seidlitz, 2018). Resting-state intrinsic brain synchronization is also supported by related gene expression (Richiardi et al., 2015). Combing neuroimaging and gene transcripts provides insights into how disease-related aberrance at the microscale architecture drives macroscale brain abnormalities in mental disorders such as depression and schizophrenia (Romero-Garcia et al., 2020; Li and Seidlitz, 2021). The details of the underlying transcriptional mechanisms of brain age remain unknown.

The aims of the current study were twofold. Firstly, we investigated the importance of brain networks in brain age prediction. The Southwest University Adult Lifespan Dataset (*N* = 492) was used in the current study. For each subject, the gray matter volumes (GMV) quantified by voxel-based morphometry (VBM) of brain regions were treated as features to predict an individual’s brain age. In the prediction model, Gaussian process regression (GPR) was chosen for its superior performance compared to existing methods (Han et al., 2021). The importance of a distinct brain network was determined by computational lesion analysis (Feng et al., 2018). Secondly, genetic annotation of the brain networks playing decisive roles in brain age prediction was generated by employing the Brain Annotation Toolbox (BAT) (Liu et al., 2019) followed by functional enrichment analysis to infer the ontological pathways of the brain age-related genes.

## Materials and Methods

### Sample

The dataset used in the current study come from the Southwest University Adult Lifespan Dataset (SALD). This dataset was obtained from healthy participants (*N* = 492, 308 females and 187 males; age range, 19–80 years). The exclusion criteria included MRI-related exclusion criteria, current psychiatric/neurological disorders, and use of psychiatric drugs in the past 3 months prior to scanning, among others. More description on the subjects and data acquisition parameters can be found in Wei et al. (2018). The data are available for research purposes through the International Neuroimaging Data-Sharing Initiative.^1^

### Data Acquisition

High-resolution T1-weighted anatomical images of the participants were acquired using a magnetization-prepared rapid gradient echo (MPRAGE) sequence (repetition time = 1,900 ms, echo time = 2.52 ms, inversion time = 900 ms, flip angle = 90°, resolution matrix = 256 × 256, slices = 176, thickness = 1.0 mm, and voxel size = 1 mm^3^ × 1 mm^3^ × 1 mm^3^).

### Voxel-Based Morphometry Analysis

We followed the standard pipeline of the CAT12 toolbox^2^ to calculate the VBM. The main steps included bias field correction, segmentation [gray and white matter and cerebrospinal fluid, adjustment for partial volume effects, normalization into the Montreal Neurological Institute (MNI) space, resampled to 1.5 mm × 1.5 mm × 1.5 mm], and non-linear modulation (Ashburner, 2009). Finally, the gray matter (GM) maps were smoothed using 6 mm full width at half maximum (FWHM) Gaussian kernel. The total intracranial volume (TIV) of each participant was also calculated to explore its association with brain age.

### Prediction Model

GPR was used to infer an individual’s brain age from the mean GMV of 246 brain regions (Fan et al., 2016) due to its superior performance (Han et al., 2021). The GPR method used in this study was implemented in the Gaussian Processes for Machine Learning (GPML) toolbox.^3^ As done in previous study (Marquand et al., 2016; Rasmussen and Williams, 2005), the parameters were optimized using a conjugate gradient optimizer (included in the GPML toolbox).

### Model Validation

A 10-fold cross-validation was used to evaluate the performance of the prediction model (Sone et al., 2019; Ziegel, 2010). This procedure was repeated 100 times to obtain more stable results. To evaluate the performance of the prediction model, we calculated (1) the mean absolute error (MAE) between the estimated brain age (output of the prediction model) and the chronological age and (2) the correlation between the chronological age and the estimated brain age across 100 repetitions. The mean brain-PAD score of each subject was calculated (brain-PAD score: predicted age - the chronological age).

To explore whether there was gender difference in the brain-PAD score, the brain-PAD scores of male subjects were compared with those of female subjects using a two-sample *t*-test controlling for age and age^2^. The correlation between the TIV and brain-PAD was also calculated to investigate its effect on brain age.

### Computational Lesion Prediction

As done in a previous study, lesion prediction analysis was performed to examine the importance of the brain networks defined in the 17 networks of Yeo et al. (2011). Specifically, the regions belonging to one specific network were excluded and the GMV of the rest of the networks were treated as features to predict brain age (Feng et al., 2018). Afterward, the importance of an individual network was determined by comparing the performance of a “lesioned” model with that of a model with all regions using Steiger’s *Z* (Feng et al., 2018; Ren et al., 2021). Here, we used the opposite value of the *Z* value. A higher *Z* meant a lower of performance of the “lesioned” model, thus declaring the more important role of the “lesioned” network in brain age prediction. The correlation between the chronological age and the mean GMV of each network was also calculated.

### Genetic Annotation Using BAT

Then, we performed a genetic annotation analysis for the brain age-related networks to identify the gene expression profile for this network using BAT^4^ (Hawrylycz et al., 2012). The gene profiles used in BAT (see text footnote 4) come from the Allen Human Brain Atlas (AHBA)^5^ obtained from six adult human brains (Hawrylycz et al., 2012). The number of anatomic samples obtained for each brain varied from 363 to 946. Details on the processing expression data were included in Liu et al. (2019). Here, we just provide a brief description. Processing the raw expression data followed the pipeline provided by the AHBA. The probe with the highest average expression was picked to represent that gene. In sum, 3,695 unique anatomic samples with 20,738 gene expression profiles were obtained. Expressions were normalized by extracting the median of the gene’s expression across all samples of the individual, then divided by the median. For each AHBA tissue sample, a 6-mm sphere region of interest (ROI) in the MNI volume space centered on its MNI centroid coordinate. Finally, 3,695 ROIs with their corresponding normalized gene expression profiles were used in the following analysis (Hawrylycz et al., 2012).

For each background AHBA sample, that with more than 50% of voxels that were also present in the given background mask was mapped to one of the given clusters. The gene expression profile of each cluster was defined as the average gene expression of all the samples mapped to the given cluster. Permutation analysis was adopted to identify the differentially expressed genes in the given cluster. Lastly, for each gene, the name and the corresponding *p*-value were obtained. In the current study, brain age-related genes were identified if their *p* < 0.05 [family-wise error (FWE) corrected] (Hawrylycz et al., 2012).

### Enrichment Pathways Associated With Brain Age-Related Genes

Thereafter, we aligned the Gene Ontology (GO) and Kyoto Encyclopedia of Genes and Genomes (KEGG) pathways with the genes obtained in the previous step using Metascape. Metascape provided automated meta-analysis tools to understand either common or unique pathways in 40 independent knowledge bases (Zhou et al., 2019). The gene list was input into the Metascape website and the results corrected by the false discovery rate (FDR; *p* < 0.05).

## Results

### Demographic Information

Demographic information of the dataset used in the current study is included in Table 1.

**TABLE 1:** Demographic information of the dataset.

	**Subjects**
Age (years), mean ± SD, (range), y	45.10 ± 17.43, (19–80)
Gender, male: female	186: 306

### Performance of the Prediction Model

**FIGURE 1:**
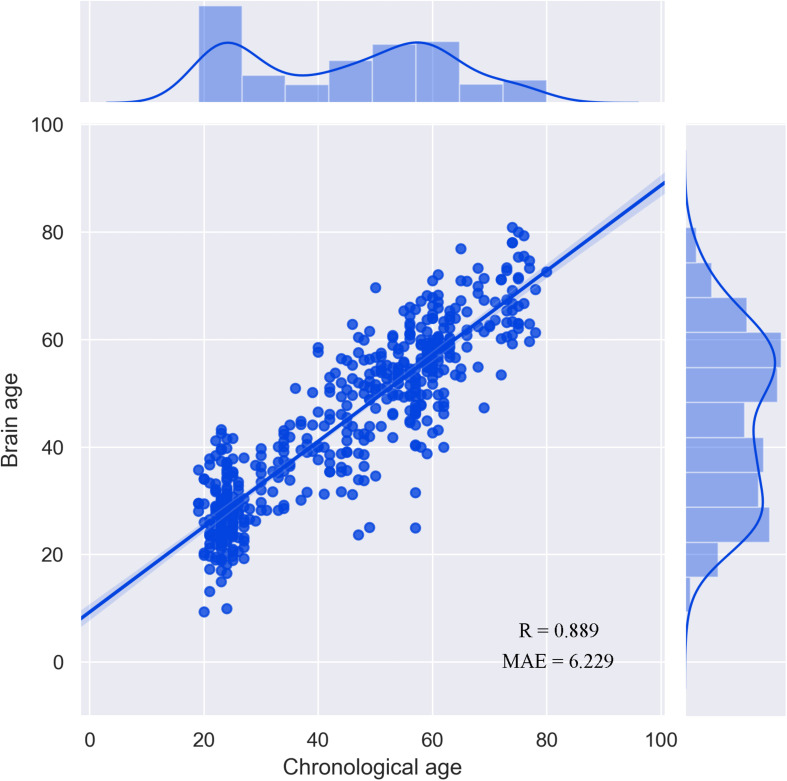
Performance of the prediction model.

The correlation between the chronological age and the estimated brain age reached *R* = 0.889 (Figure 1). Consistent with the findings of a previous study, the performance of the prediction model was better that that in Han et al. (2021) because the sample size used in the current study was larger (Franke et al., 2010). There was no significant difference between male and female subjects (*p* > 0.05). The correlation between TIV and brain-PAD was also not significant (*p* > 0.05).

### Computational Lesion Prediction

The results of computational lesion prediction revealed that the performance of the prediction model significantly degraded (Steiger’s *Z* = 2.114, *p* = 0.035) only if the central executive network, including the bilateral middle temporal gyrus, right middle frontal gyrus, the bilateral dorsolateral frontal gyrus, and the right inferior parietal lobule, was excluded (Supplementary Figure 1). The mean GMV of the 17 networks were all negatively correlated with the chronological age, suggesting that the GMV decreases in normal aging (Supplementary Figure 2).

### Enrichment Pathways

**FIGURE 2:**
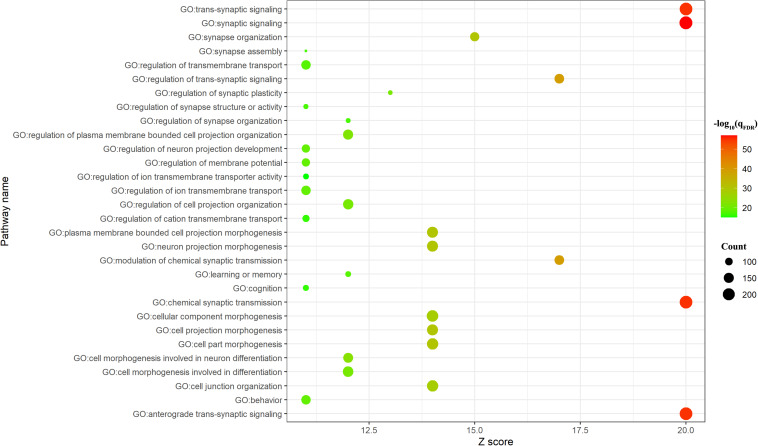
Top 20 significant Gene Ontology (GO) biological processes/Kyoto Encyclopedia of Genes and Genomes (KEGG) pathways. The count meant the number of genes involved in the given term.

**FIGURE 3:**
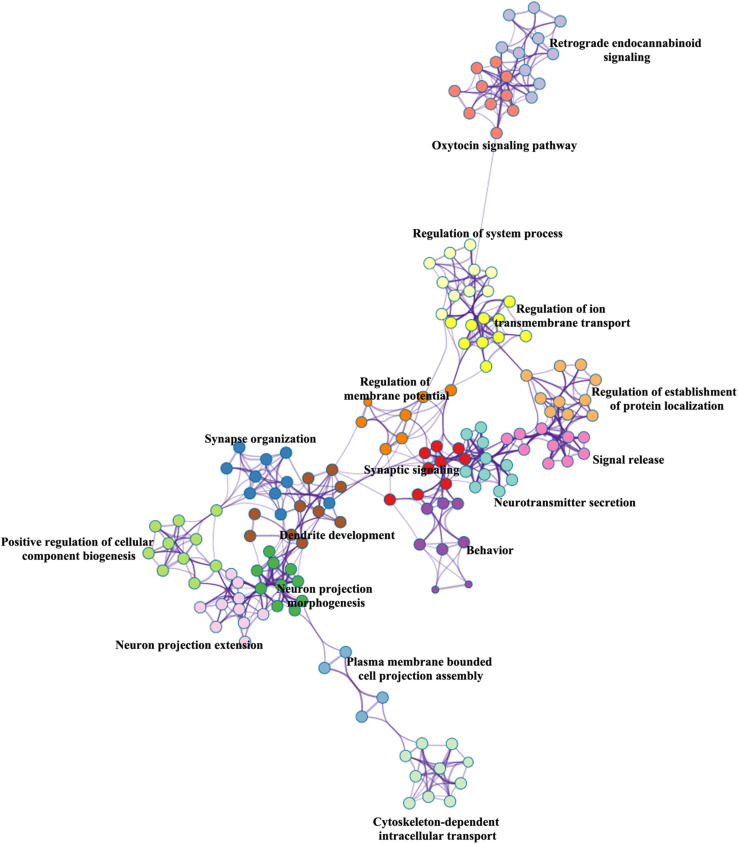
Metascape enrichment network visualization.

BAT identified 2,927 genes associated with brain age-related networks. Then, we aligned the GO biological processes and KEGG pathways using Metascape. The results reported in this study were corrected for FDR (*p* < 0.05) and discrete enrichment clusters were discarded. The GO processes and KEGG pathways were clustered into a number of groups such as regulation of iron transmembrane transport, synaptic signaling, synapse organization, retrograde endocannabinoid signaling (e.g., dopaminergic synapse), behavior (e.g., memory and associative learning), neurotransmitter secretion, and dendrite development. The top 20 enrichment terms were included in Figure 2 and the enrichment networks were drawn in Figure 3.

## Discussion

In this study, we investigated the importance of brain networks contributing to brain age prediction and the underlying molecular mechanisms of brain age. As a result, the central executive network turned out to be a vital network in predicting brain age due to the performance of the prediction model being significantly degraded (Steiger’s *Z* = 2.114, *p* = 0.035) when it was excluded from the model. The genes associated with the central executive network were ontologically enriched in clusters such as regulation of ion transmembrane transport, synaptic signaling, synapse organization, retrograde endocannabinoid signaling (e.g., dopaminergic synapse), behavior (e.g., memory and associative learning), and so on. In all, these results reveal that the GMV of the central executive network played a vital role in predicting brain age and bridged the gap between transcriptome and neuroimaging promoting an integrative understanding of the pathophysiology of brain age.

Our results hinted that the GMV of the central executive network is a potential biomarker of brain age. Normal brain aging is associated with GM volume loss (Allen et al., 2005; Walhovd et al., 2005), including in the parietal lobe, temporal cortex, and especially in the frontal lobe (Matsuda, 2013; Van Petten et al., 2004). Along with losses of GMV, normal aging is characterized by a gradual decline in cognitive processes such as executive function, episodic memory, working memory, and processing speed (Lee et al., 2016). Consistent with these studies, our results presented that the GMV of all networks correlated with brain age significantly. In addition, we found that only when the central executive network was excluded did the performance of the prediction model significantly degrade (Steiger’s *Z* = 2.114, *p* = 0.035). These results hinted that the central executive network could be a potential biomarker of brain age. The reason might be that the effect of brain aging on the central executive network was more consistent across different populations than regions like the amygdala, hippocampus, and thalamus (Matsuda, 2013). Individuals exhibiting age-related decline tended to show impairments of executive functions first, suggesting that this network might be particularly vulnerable during normal aging (Sorel and Pennequin, 2008). In addition, a linear volume reduction of the central executive network with increasing age even occurred during the earlier stages of adulthood (Terribilli et al., 2011). As a supplement to these studies, our results revealed that the GMV of the central executive network played a decisive role in predicting brain age.

We further investigated the transcriptional signatures of the brain age-related networks. Although brain age was employed in abnormal aging trajectories in various diseases (Gaser et al., 2013; Habes and Janowitz, 2016; Hajek et al., 2019; Han et al., 2021; He et al., 2020), studies investigating the underlying biological foundation of brain age are scarce. To the best of our knowledge, only one study linked polygenic risk score and accelerated brain aging in AD (Habes and Janowitz, 2016). For the first time, we found that brain age-related genes were enriched in GO processes/KEGG pathways clustered into a number of groups such as regulation of iron/calcium transmembrane transport, synaptic signaling, synapse organization, retrograde endocannabinoid signaling (e.g., dopaminergic synapse), behavior (e.g., memory and associative learning), neurotransmitter secretion, and dendrite development. Calcium-dependent signals were key triggers of the molecular mechanisms underlying learning and memory; dysregulation of its homeostasis in the aging brain was hypothesized to underlie aging-related cognitive decline (Oliveira and Bading, 2011). In the brain, iron was involved in many fundamental biological processes, including neurotransmitter synthesis and metabolism; its homoeostasis played an important role in maintaining normal function (Ward et al., 2014). Normal brain aging is accompanied by selective accumulation of iron. Greater accumulation of iron was observed in neurodegenerative diseases associated with oxidative stress and cellular damage (Zecca et al., 2004). In addition, both the density and morphology of dendritic trees mainly possessed by pyramidal neurons underwent progressive regression in the neocortex (Dickstein et al., 2013) without neuronal death (Morrison and Hof, 1997). Consistent with the notion that no single mechanism explains the aging process (Kyng et al., 2003), we identified a number of GO processes/KEGG pathways underlying brain age.

Several limitations should be considered when understanding our results. Firstly, factors such as educational level could also affect the GMV. For example, greater GMV in the superior temporal gyrus, insula, and anterior cingulate cortex were found in more educated individuals (Arenaza-Urquijo et al., 2013). As this information was not included in the dataset used in the current study, future studies might explore its effect on brain age. Secondly, the gene expression data and neuroimaging data did not come from the same subjects. Considering the high degree of conservation in overall gene expression across human populations (Stranger et al., 2007; Zhu et al., 2021), the expressions of brain age-related genes could be believable.

## Conclusion

As a supplement to previous studies exploring brain age, our results reveal a decisive role of the GMV of the central executive network in brain age prediction. In addition, the present study investigated the underlying transcriptional profiling of the central executive network. As a result, we found that brain age-related genes were enriched in GO processes/KEGG pathways clustered into a number of aging-related mechanisms such as regulation of iron/calcium transmembrane transport and dendrite development. In all, these results reveal that the GMV of the central executive network played a vital role in predicting brain age and bridged the gap between transcriptome and neuroimaging promoting an integrative understanding of the pathophysiology of brain age.

## Data Availability Statement

The original contributions presented in the study are included in the article/Supplementary Material, further inquiries can be directed to the corresponding author/s.

## Ethics Statement

The studies involving human participants were reviewed and approved by the Research Ethics Committee of the Brain Imaging Center of Southwest University, in accordance with the Declaration of Helsinki. The patients/participants provided their written informed consent to participate in this study.

## Author Contributions

KF analyzed the data and wrote the manuscript. SH designed the research, analyzed the data, and wrote the manuscript. YL and JD searched the literature. JW modified the language. WZ directed the research program and provided guidance and suggestions for the study. All authors read and approved the final manuscript.

## Conflict of Interest

The authors declare that the research was conducted in the absence of any commercial or financial relationships that could be construed as a potential conflict of interest.

## Publisher’s Note

All claims expressed in this article are solely those of the authors and do not necessarily represent those of their affiliated organizations, or those of the publisher, the editors and the reviewers. Any product that may be evaluated in this article, or claim that may be made by its manufacturer, is not guaranteed or endorsed by the publisher.

## Footnotes

1
http://fcon_1000.projects.nitrc.org/indi/retro/sald.html

2
http://dbm.neuro.uni-jena.de/cat12/

3
www.gaussianprocess.org/gpml/code/

4
http://123.56.224.61/softwares

5
http://human.brain-map.org/
