# Supplementary material for: The Vital Role of Central Executive Network in Brain Age: Evidence From Machine Learning and Transcriptional Signatures
Source: Front Neurosci. 2021 Sep 7;15:733316. doi: 10.3389/fnins.2021.733316 (PMC8453084; doi:10.3389/fnins.2021.733316)

**Figure S1**. Brain regions of central-executive network. Regions were labeled in labeled in different colors.


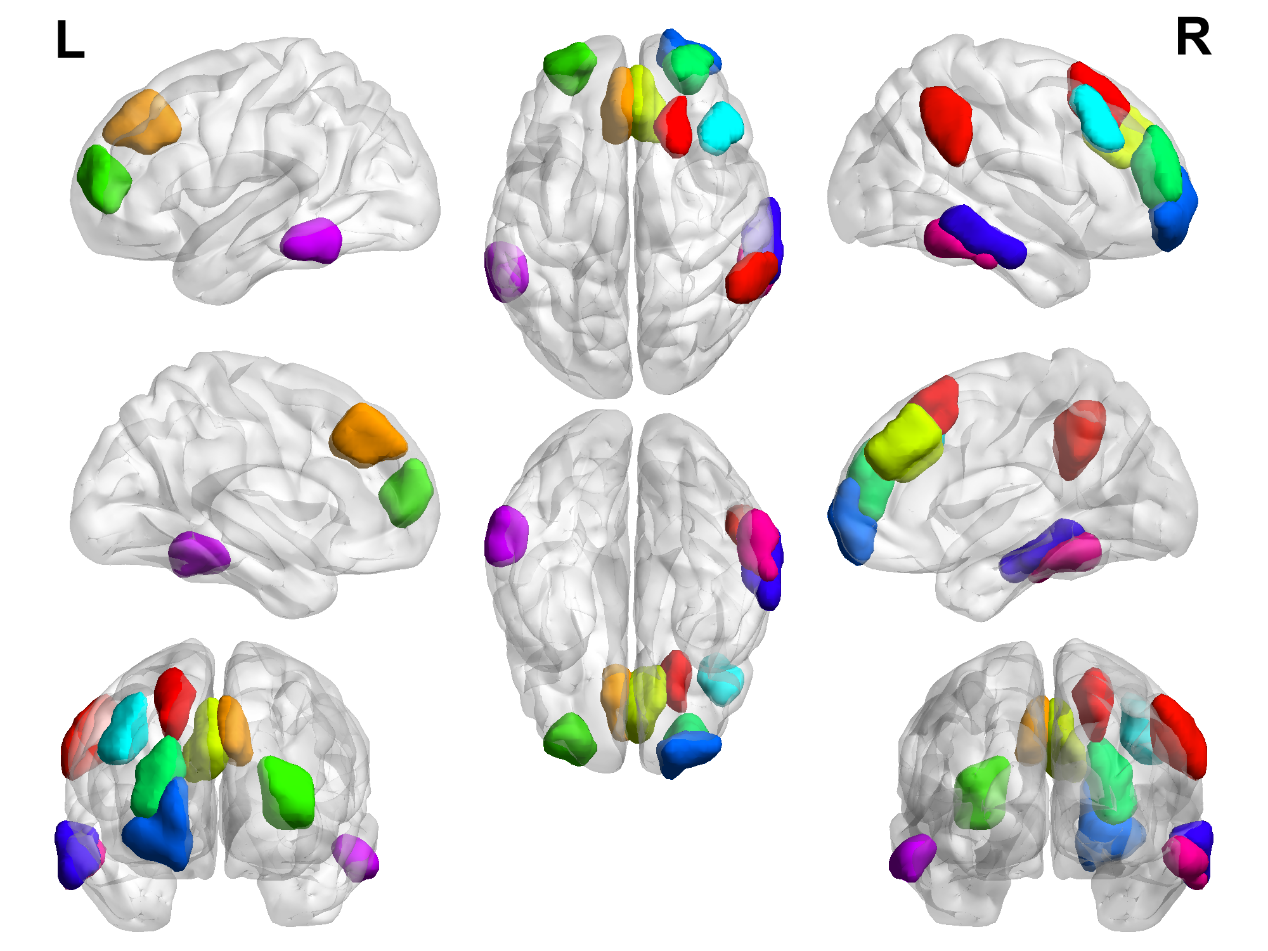


**Figure S2**. The negative correlation between the chronological age and GMV of 17 networks. The brain age related network (central-executive network) was marked with “*”.


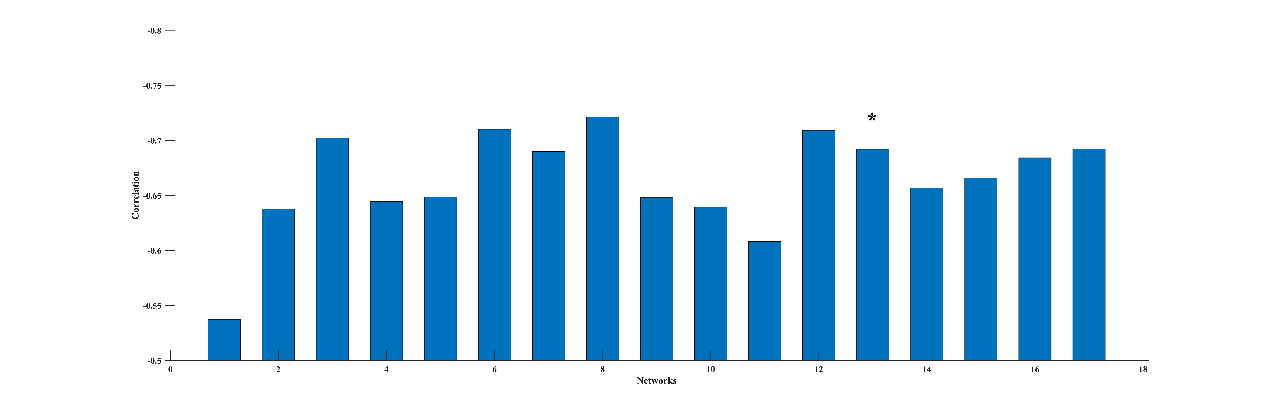

Supplement: Supplementary file 1 [file Data_Sheet_1.docx]
